# EGFR kinase domain duplication in lung adenocarcinoma with systemic and intracranial response to a double-dose of furmonertinib: a case report and literature review

**DOI:** 10.3389/fonc.2024.1321587

**Published:** 2024-06-21

**Authors:** Hong Lin, Zhengyuan Yang, Zhifeng Li, Junwei Chen, Hongbiao Wang, Yingcheng Lin

**Affiliations:** ^1^ Department of Medical Oncology, Cancer Hospital of Shantou University Medical College, Shantou, Guangdong, China; ^2^ Department of Radiology, Cancer Hospital of Shantou University Medical College, Shantou, Guangdong, China

**Keywords:** EGFR-KDD, furmonertinib, brain metastasis, lung adenocarcinoma, targeted therapy

## Abstract

**Background:**

EGFR kinase domain duplication (EGFR-KDD) is an infrequent oncogenic driver mutation in lung adenocarcinoma. It may be a potential target benefit from EGFR-tyrosine kinase inhibitors (TKIs) treatment.

**Case presentation:**

A 66-year-old Chinese male was diagnosed with lung adenocarcinoma in stage IVb with brain metastases. Next-generation sequencing revealed EGFR-KDD mutation. The patient received furmonertinib 160mg daily for anti-cancer treatment and obtained therapeutic efficacy with partial response (PR). Progression-free survival (PFS) duration from monotherapy was 16 months. With slow progressions, combined radiotherapy and anti-vascular targeted therapy also brought a continuous decrease in the tumors. The patient has an overall survival (OS) duration of more than 22 months and still benefits from double-dose furmonertinib.

**Conclusions:**

This report provided direct evidence for the treatment of EGFR-KDD to use furmonertinib. A Large-scale study is needed to confirm this preliminary finding.

## Introduction

Global cancer statistics demonstrate that lung cancer remains the leading cause of cancer-related deaths worldwide (1). Adenocarcinoma is the most common pathological type of lung cancer, accounting for 35-40% of all cases. Patients with oncogenic mutations of the epidermal growth factor receptor (EGFR) tyrosine kinase domain have been identified as a significant subgroup of non-small cell lung cancer (NSCLC). Compared with standard chemotherapy, EGFR tyrosine kinase inhibitors (TKIs) generally obtain better tumor control in patients with EGFR-mutated lung adenocarcinoma (2, 3). The most common EGFR-activating mutations include in-frame deletions in exon 19 (Ex19del) and point mutations in exon 21 (Eex21 L858R), which are generally sensitive to EGFR-TKIs (4). EGFR kinase domain duplication (EGFR-KDD) represents a rare form of EGFR mutation, with an incidence of 0.24% in EGFR mutation of lung cancer (5). Predominantly, EGFR-KDD is caused by in-frame tandem duplication of EGFR exons 18–25. Aberrant duplication forms an intramolecular dimer and constitutively activates EGFR signaling. Some studies also reported duplications spanning exons 17 to 25 or exons 14 to 26 (6). Several reports have demonstrated significant anti-tumor responses to EGFR TKI treatment for lung adenocarcinoma patients with EGFR-KDD alterations, including gefitinib, erlotinib, afatinib, and osimertinib (7–9). However, there is currently no standardized approach for treating EGFR-KDD alterations. Here, we present a case report of a patient with harboring EGFR-KDD alteration and brain metastases. The patient is treated with furmonertinib and obtains an optimal cancer response duration. This is the first case report of furmonertinib use in EGFR-KDD alterations.

## Case report

**Figure 1:**
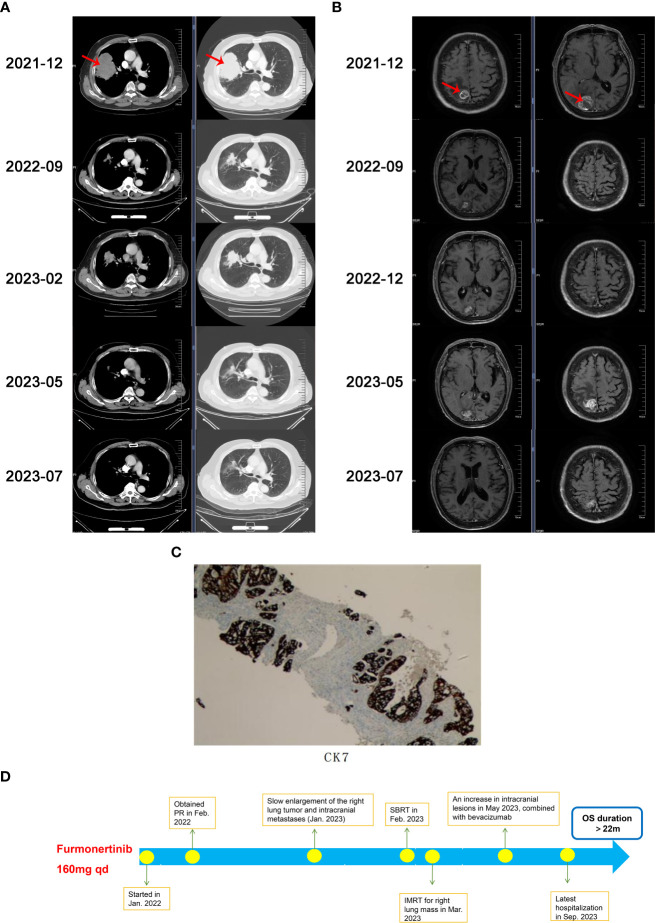
**(A)** Chest computed tomography (CT)scans during the treatment, **(B)** Cerebral magnetic resonance imaging (MRI)scans during the treatment, **(C)** Immunohistochemical image of CK7, **(D)** The timeline demonstrating the history of treatment for the patient. PR, partial response; SBRT, stereotactic body radiation therapy; IMRT, intensity-modulated radiation therapy; OS, overall survival.

A 66-year-old Chinese male presented with lower limbs weakness and was admitted to a local hospital. Cerebral computed tomography (CT) scan revealed a right occipital lobe mass. Further positron emission tomography-CT (PET-CT) imaging revealed that F-18 fluorodeoxyglucose hypermetabolic speckled in a large lesion in the right lung, suggesting of peripheral lung cancer with brain metastasis. In December, 2021, the patient transferred to our hospital. He was previously diagnosed with hypertension, and has no family history of malignancy. The Eastern Cooperative Oncology Group (ECOG) score of the patients was 1. Physical examination showed sightly attenuated breath sounds in the right lung. A chest CT scan revealed a 10 x 8 cm mass in the upper right lung and enlarged lymph nodes in the mediastinum (**Figure 1A**). Magnetic resonance imaging (MRI) demonstrated metastatic masses in the right parietal and occipital lobes measuring 2 x 1.8 cm and 3.2 x 3.1 cm (**Figure 1B**), respectively, as well as metastases in the second and third sacral vertebra. CT-guided biopsy of the right lung mass was performed and showed adenocarcinoma on pathology (**Figure 1C**). According to the 8th edition of AJCC staging, the patient was classified as stage IVb (cT4N2M1c). Next-generation sequencing (NGS) revealed an EGFR-KDD mutation involving exons 18-25 (frequency 18%), accompanied by TP53 D281F indel (frequency 9%). The patient received furmonertinib at a double dose of 160 mg for targeted therapy in January 2022, in order to increase drug concentration in the brain tissue. Following treatment, the patient’s bilateral lower limb weakness gradually ameliorated, and he regained the ability to walk independently. One month later, a chest CT scan revealed that the right lung tumor had shrunk from 10 cm to 5.7 cm. Subsequent regular follow-up examinations revealed that the best therapeutic efficacy was partial response (PR). The right lung tumor shrunk to 4.2 x 2.6 cm (in September, 2022), and the brain lesions also significantly decreased in size. Images of CT and MRI scans were showed in **Figures 1A, B**. Importantly, the patient showed good tolerance to the double dose of furmonertinib without diarrhea, liver function injury, interstitial lung disease or other adverse reactions. There was no reduction or discontinuation of furmonertinib due to any adverse events regarding to double dose.

In December 2022, a chest CT scan and cerebral MRI showed slow progression of the right lung tumor and intracranial metastases, but did not meet the criteria for diagnosing progressive disease (PD). The patient refused the suggestion of biopsy to clarify secondary mutations and continued to receive furmonertinib at a daily dose of 160mg. In January 2023, the patient experienced bilateral lower limb weakness again and received stereotactic body radiation therapy (SBRT) for the brain metastases. In February 2023, a follow-up CT scan revealed further enlargement of the right lung tumor, and the patient underwent intensity-modulated radiation therapy (IMRT) for the lesion in the right lung and the surrounding lymph drainage area.

In May 2023, the patient suffered from lower limbs weakness again. A cerebral MRI showed an increase in intracranial lesions with peritumoral edema, while a chest CT scan showed that right lung tumor continued to shrink, maintaining a PR evaluation. Following multidisciplinary discussions, the recommended treatment was surgical resection of the intracranial metastases. However, the patient refused surgery. In order to further enhance intracranial drug concentrations and reverse tumor resistance, we added bevacizumab at a dose of 7.5mg/kg every three weeks as anti-vascular targeted therapy, starting from May 17, 2023. Furmonertinib has been continuously administered up to now. The lower limbs weakness improved, and multiple reassessments indicated a PR treatment evaluation. Since the diagnosis, the patient has achieved a progression-free survival (PFS) duration of 16 months with first-line monotherapy of furmonertinib, and currently continues to benefit from furmonertinib, with an overall survival (OS) duration exceeding 22 months. The patient has not suffered from significant adverse drug reactions during the treatment. The flowchart of treatment is showed in **Figure 1D**.

## Discussion

Exon 19 deletion and exon 21 L858R mutation are the two most common EGFR mutations, accounting for 75% of all EGFR mutations and representing the sensitive mutations for EGFR-TKI treatment (10). EGFR-KDD is a rare mutation. Wang et al. (5) reviewed 10759 cases of lung cancer and found that EGFR-KDD accounted for only about 0.24% of EGFR mutations. As a driver gene, EGFR-KDD mutations are commonly observed in exons 18-25, with exon 17-25 and exon 14-26 being less common. Vitro studies have shown that repetitive mutation in EGFR exons 18-25 kinase domain lead to constitutive activation of the EGFR kinase, resulting in over-activation of downstream signaling pathways, promoting cell proliferation and tumor development. Additionally, clinical pathological studies reported that EGFR-KDD alteration may increase sensitivity to EGFR-TKIs (8, 11). However, there is currently no consensus on the treatment of EGFR-KDD. Previous publications about EGFR-KDD treatments are mostly case reports, with EGFR-TKI being the choice for most first-line treatments, although the treatment is not as effective as EGFR sensitive mutations. Yang et al. (12) reported a case of an EGFR-KDD mutant patient who received chemotherapy with unsatisfactory results. **Table 1** summarizes the published cases of EGFR-KDD mutation treating with first-line TKIs. The longest reported PFS is 12 months, although patients still benefit from targeted therapy at the time of publication. The only patient with brain metastasis experienced disease progression after only 2 months of treatment with osimertinib.

**Table 1:** Clinical characteristics and outcomes of patients with NSCLC EGFR-KDD mutation treating with first-line TKIs in previous studies.

No.	Publication	Year of publication	Age	Gender/ Ethnicity	Diagnosis/ Stage	EGFR-TKI Treatment	Concurrent mutations	Response to TKI	PFS (months)
1	Zhu et al. (13)	2018	63	Female/ Chinese	LUAD/IV	Icotinib	NA	SD	11
2	Chen et al. (14)	2020	59	Male/ Chinese	LUAD/IV	Afatinib	TP53 R282W CTNNB1 S37Y	SD	10
3	Zhao et al. (11)	2021	61	Male/ Chinese	LUAD/IIIB	Afatinib	None	PR	12
4	Kim et al. (15) *	2021	50	Male/ African- American	LUAD/IVb	Osimertinib	None	PR	2
5	Wang et al. (16)	2019	74	Female/ Chinese	LUAD/IIb	Afatinib	None	SD	6
6	Zhang et al. (9)	2021	#1:44	#1:Male/ Chinese	#1:LUAD/ IVa	#1: Afatinib→ Osimertinib	#1: CDK6 TP53	#1: PR SD	#1: 7(1^st^ line) NR(2^nd^ line)
			#2:61	#2:Female/Chinese	#2:LUAD/ IVb	#2:icotinib	#2:PTEN	#2:NR	#2:8
7	Wang et al. (5)	2019	#1:61 #2: 60 #3: 67 #4: 43 #5: 52	#1: Male/ Chinese #2: Male /Chinese #3: Female/ Chinese #4: Male/ Chinese #5: Female/ Chinese	#1: LUAD/IV #2: LUAD/IV #3: LUAD/IV #4: LUAD/IV #5: LUAD/IV	#1: Erlotinib→ Osimertinib #2: Gefitinib→ afatinib→ osimertinib #3: Gefitinib #4: Icotinib + patinib #5: Gefitinib→ erlotinib	#1: TP53 #2: ERBB2 #3: NA #4: TP53, PIK3CA #5: NA	#1: PD #2: PR #3: SD #4: PR #5: PD	#1: 2(1^st^ line) 2 (2^nd^ line) #2: 5(1^st^ line), 2(2^nd^ line), 4(3^rd^ line) #3: 11 #4: 4 #5: 3(1^st^ line) 5(2^nd^ line)

* accompanied with brain metastasis; #: a report with multiple patients

NSCLC, non-small cell lung cancer; EGFR-KDD, epidermal growth factor receptor kinase domain duplication; TKI, tyrosine kinase inhibitor; PFS, progression-free survival; LUAD, lung adenocarcinoma; NA, not available; SD stable disease; PR, partial response; NR, not reach; PD, progressive disease

We are the first to report a case of EGFR-KDD mutated lung adenocarcinoma with concurrent brain metastasis treated with furmonertinib. Furmonertinib (AST2818) is a highly brain-penetrant, third generation EGFR tyrosine kinase inhibitor (TKI) developed by Shanghai Allist Pharmaceuticals Co., Ltd, Shanghai, China. It has been approved for the first-line treatment of patients with 19Del or L858R mutations and treatment of patients with T790M mutations whose disease has progressed on or after EGFR TKI therapy (17, 18). Compared with osimertinib, it has atrifluoroethoxy pyridine-based molecule structure that binds to a hollow hydrophobic pocket in the binding region composed of amino acids M793 and L792, enhancing its binding activity and kinase selectivity for EGFR. Additionally, this modification improved the metabolic profile of furmonertinib and inhibited the formation of non-selective metabolites. Furthermore, furmonertinib and its metabolites exhibit low affinity for wild-type EGFR-encoded proteins, which minimizes inhibition of wild-type EGFR-encoded proteins and reduces related side effects, thus improving its safety. Meng et al. revealed that furmonertinib is mainly distributed in the lung after administration (19). Therefore, furmonertinib improve treatment efficacy of lung cancer. In the FAVOUR (NCT04858958, CTR20201697) phase Ib study, furmonertinib 240 mg once daily and 160 mg once daily both showed promising efficacy and a predictable and manageable safety profile in patients with NSCLC harboring EGFR Exon20ins mutations (20). Furmonertinib might play a role in the treatment of rare EGFR mutation. A pooled, *post-hoc* analysis of two phase 2 studies (NCT03127449 [phase 2a study of furmonertinib], NCT03452592 [phase 2b study of furmonertinib]) demonstrated that better response and longer median central nervous system-PFS (CNS-PFS) were observed in patients treated by furmonertinib 160 mg orally once daily to furmonertinib 80mg (21). Regarding the safety of medication use, FUTONG study indicated that furmonertinib and gefitinib have similar rates of drug-related adverse reactions at standard doses. However, for grade 3 or higher adverse reactions, the incidence in the furmonertinib group (18%) was lower than that in the gefitinib group (11%) (18). The most frequent severe adverse reactions with furmonertinib are QTc prolongation and diarrhea. Furthermore, a retrospective single-arm study presented at the 2023 World Conference on Lung Cancer (WCLC) demonstrated that out of 20 patients with advanced non-small cell lung cancer (NSCLC) harboring EGFR mutations and brain metastases treated with a first-line double dose of furmonertinib, only one patient experienced grade 3 treatment-related adverse events (TRAEs) (22). Three patients had their dosage reduced, and one patient interrupted treatment due to TRAEs, but no patients discontinued the study medication. These findings suggest that a double dose of furmetinib also exhibits a favorable tolerability profile. In this case, the patient has not experienced significant adverse reactions during treatment, demonstrating great safety of furmonertinib. Furmonertinib may emerge as a new option for treating EGFR-KDD mutation.

TP53 is a critical tumor suppressor gene and has the highest mutation rate among malignancies. In NSCLC patients, 50%-65% of EGFR mutation-positive cases also harbor TP53 mutations. Clinical research has consistently shown that TP53 mutations impact the effectiveness of EGFR-TKIs. A meta-analysis including 24 studies with 2,227 patients with EGFR-mutated NSCLC found that patients with TP53-EGFR co-mutations had significantly shorter PFS and overall survival (OS) duration compared to those with wild-type TP53 (23). Subgroup analysis indicated that mutations in exons 5-8 were associated with a poorer prognosis. Further research has found that in advanced EGFR-mutated NSCLC patients, the presence of TP53 exon 4 or 6 mutations leads to an even worse outcome (24). Although the effect of specific TP53 mutation subtypes on prognosis is not fully agreed upon, the co-occurrence of TP53 and EGFR mutations generally decreases the efficacy of EGFR-TKIs in NSCLC patients. Some studies have attempted to explore the effects of combination therapy. The RELAY study (25), a phase III trial, investigating the efficacy of ramucirumab plus erlotinib versus placebo plus erlotinib in EGFR-positive NSCLC patients, showed that patients with TP53 co-mutations at baseline benefited more from combination therapy in terms of PFS, regardless of exon 19del or exon 21 L858R mutations. The ACTIVE study (26) suggested that a combination of gefitinib and apatinib favored PFS for patients with TP53 mutations compared with gefitinib alone, particularly for those with exon 8 mutations. Additionally, a retrospective study (27) demonstrated that the combination of EGFR-TKI with chemotherapy could provide more survival benefits than EGFR-TKI monotherapy for NSCLC patients with the TP53-EGFR co-mutation. Despite the potential benefits, combination therapy also increases the rate of adverse reactions, which may be challenging for patients to tolerate. In our case, the patient had a TP53 exon 8 D281F mutation, which could be a negative prognostic factor. However, the patient still achieved satisfactory tumor control with monotherapy of furmonertinib.

Previous studies demonstrated that continuing the original TKI treatment in combination with consolidative local therapy benefits patients with central progression or oligoprogression after targeted therapy (28). Furthermore, multiple studies suggested that the combination of targeted therapy and anti-angiogenesis treatment significantly improves PFS (29, 30). The approach of anti-angiogenesis plus targeted therapy is a crucial component of chemotherapy-free treatment strategies. The combined therapy simultaneously blocks the EGFR/VEGF pathway and downregulates signaling pathways at multiple sites, exhibiting synergistic anti-tumor activity and delaying the occurrence of TKI resistance. Additionally, the vessel normalization effect of anti-angiogenic agents alleviates the impact of the blood-brain barrier, improving drug transport in the central nervous system and increasing intracranial drug concentrations. In this case, the patient experienced slow enlargement of intracranial and right lung lesions after 12 months of furmonertinib use. After receiving local radiotherapy, the tumor continued to shrink. In May 2023, the tumor progressed again, and the patient declined further local interventions. We added bevacizumab to the furmonertinib and the patient obtained persistent tumor control. As of now, the patient continues to benefit from the combination treatment approach, with an OS duration exceeding 22 months.

In summary, we report the first case of furmonertinib using in advanced non-small cell lung cancer (stage IVb) with an EGFR-KDD mutation. The patient achieved a sustained anti-tumor response in primary tumor and central nervous system metastases with a double-dose of furmonertinib. Compared to first-generation and second-generation EGFR TKIs, furmonertinib has better penetration across the blood-brain barrier and demonstrates efficacy in treating central nervous system metastases in non-small cell lung cancer, showing a favorable response rate. In comparison to other third-generation EGFR TKIs such as osimertinib, furmonertinib has fewer adverse reactions and higher safety, which bring better patient compliance. This case report supports the use of furmonertinib in advanced NSCLC patients with EGFR-KDD and central nervous system metastasis. Large scale study is needed to confirm this preliminary finding.

## Data availability statement

The raw data supporting the conclusions of this article will be made available by the authors, without undue reservation.

## Ethics statement

Written informed consent was obtained from the individual(s) for the publication of any potentially identifiable images or data included in this article.

## Author contributions

HL: Formal analysis, Methodology, Software, Writing – original draft. ZY: Data curation, Formal analysis, Methodology, Writing – original draft. ZL: Formal analysis, Methodology, Writing – original draft. JC: Conceptualization, Project administration, Supervision, Writing – review & editing. HW: Conceptualization, Investigation, Supervision, Writing – review & editing. YL: Supervision, Visualization, Writing – review & editing.
